# Institutional Notes and News

**Published:** 1910-10-15

**Authors:** 


### Institutional Notes and News.

The new cottage hospital at Castle Mount, Bangor, Ireland, has been opened by Mr. W. K. Crosby. Lady Clanmorris, who presided, reminded those present at the ceremony that the first cottage hospital in Ireland was opened there by her mother many years ago. The medical and surgical wards are eight in number, the original cottage hospital, which has done good work for forty years, being now too small to meet the demands of Bangor's growing population. The site has been given rent free during the lives of Lord and Lady Clanmorris and the next heir of the estate, and of the £4,000 which the hospital has cost, all but £400 has been subscribed. Sir John Byers, M.D., and Professor Lindsay also spoke. The architects are Messrs. Young and Mackenzie, Belfast.

**Bangor Cottage Hospital.**

An interesting account of how a valuable bequest came from a printer to the Surrey County Hospital has been given by Mr. Herbert A. Powell, its treasurer, in a letter to a local newspaper, from which we take the following: "John Wray (the printer) was born in the South of London in 1821. At the age of twenty-one he apprenticed himself for seven years to a member of the Stationers' Company in London. It is uncertain in which year he came to Guildford, but in 1867 he was 'a printer at Mr. Billing's office there.' It is interesting to recall that it was in this year that the County Hospital was being built. At the instance of Mary Wray, who was two years older than her brother, a friend invested for him in Consols about £100, which had been sewn up in his sister's bed, and a further sum which had been placed in a Trustee Savings Bank. These amounts were the result of twenty-two years' thrift; for it is on record that he put his first sovereign in a savings-bank in 1845, after being apprenticed for three years. He eventually returned to Blackfriars, and underwent an operation at the Royal Westminster Ophthalmic Hospital, and among his papers were found receipts for a donation of £3 3s., and subsequent annual subscriptions of 10s. to its funds. After his retirement he lived on the income from his invested savings, reinforced by an allowance from the Stationers' Company, and died at the age of seventy-eight. Subject to the life-interest of his sister, who has just died in her ninety-first year, he left the whole of his accumulated savings to be divided in certain proportions between the Surrey County Hospital and the Printers' Pension Corporation. The share of the hospital amounts to £374 7s. 3d." This is a striking example of the influence hospitals often exert even on the hearts of people fortunate enough to need their help rarely.

**Surrey County Hospital.**

We are glad to be able to state that nobody was injured in the rather serious fire that destroyed part of the east wing of the Leeds General Infirmary on Friday morning in last week. Every member of the staff is to be congratulated on the order which prevailed; there was no panic and no excitement, and no one was any the worse for the experience. Soon after 7 A.M. on October 7 a passer-by noticed smoke rising from the roof before anyone in the children's ward knew about it. He gave notice to the night porter, who summoned the fire brigade, while successful efforts were made by the day nurses, doctors, and wardmaids to remove the thirty-five or forty children to a place of safety, and the furniture also was quickly moved out. The wards on the two floors below, 16, 17, 14, 15, were also cleared. Thus everything was done to prevent a panic, so that the firemen might begin to work at the earliest moment. Owing to a difficulty in getting water, three hours were required before the firemen were able to get the conflagration under control, the fire spreading downwards by means of a defective flue. The flames were not put out, however, until the two children's wards Nos. 18, 19, and a small ward, No. 20, had been completely destroyed. The building is insured. The House Committee and its Chairman, Dr. Charles Lupton, and the medical staff were on the scene early, and their anxiety was shared in every part of Leeds. The east wing was built some twenty years ago at a cost of over £48,000; but the Infirmary's work, we learn, will not be interfered with, as room has been found elsewhere for those patients who were housed in the damaged wards. The damage is estimated at over £1,000. At the time of the fire there were thirty children and sixty other patients in the east wing, while there are 400 beds in the Infirmary. In the face of the splendid discipline shown on this occasion, it would be interesting to learn how often fire-drills take place at Leeds Infirmary.

**Leeds General Infirmary.**

The Mayor of Barnstaple, Mr. F. W. Hunt, C.C., presided at the recent annual meeting of the North Devon Infirmary, when Mr. C. E. R. Chanter remarked that if the institution had had the same legacies this year as it had had in 1908 the balance sheet would not be showing a deficit of £370, for this source of revenue had dropped from £450 to £15 in two years. It was satisfactory to note that the new oculist and dental departments had been opened, the former being much patronised, and that no part of the expense fell upon the general funds of the Infirmary. The furnishing was paid for by the trustees of the late Mr. W. Allen. Expenses had been cut down by closing the Fortescue Ward, which was done regretfully last March, three nurses and a maid being discharged soon afterwards, which economies resulted in a saving of £91 odd in the June quarter. On the other hand, the health of the staff had caused some anxiety. The Balcony Ward had been very much appreciated, and had been used on 147 days. During the year 754 patients had been treated in the wards, as compared with 815 for the preceding year, while 2,614, as compared with 2,904, persons had attended the out-patient department. The report of the house surgeon, Mr. L. W. Evans, stated that the average daily number of patients in the house was forty.

**North Devon Infirmary.**

In the year 1897 Sir David L. Salomons, Bart., of Broomhill, Tunbridge Wells, presented to its general hospital a very complete *x*-ray apparatus, which has since then been of much service and is still in use. On Saturday, October 8, 1910, the same gentleman again showed his interest in the institution and his care for the relief of the suffering injured and sick poor by formally giving a very powerful *x*-ray installation of the most recent pattern to the hospital authorities. The new apparatus is adapted for an alternating current, and is supplied with power from the town supply of 220 volts, with switch table, exposure regulator, pole indicator, ampmeter and voltmeter; can be used for instantaneous radiography, time exposures, and screen work, a very complete arrangement being provided for the latter. In making the presentation, Sir D. L. Salomons, Bart., briefly outlined the progress made in *x*-ray work,

**General Hospital, Tunbridge Wells.**

and the benefits which had been gained by pioneer research and at the cost of the sufferings of the earlier investigators. He regretted that the "Finsen" light which he had ordered was not yet delivered. Speaking of the discovery of radium, Sir David said that he had one thing in his pocket which he wished to hand over to the staff, if they would accept it. That was a little radium, fixed as is usual upon a disc, varnished, fitted to a handle, and arranged so that various areas of the disc might be applied as required. Sir David suggested that its use should not be confined to the hospital, but that it should be lent to practitioners when desirable, the patients, if able, paying a small fee. The Mayor, Mr. F. Wadham Elers, J.P., who is also Chairman and Treasurer of the hospital, heartily thanked Sir David for his address and gifts, and he was quite certain that the Committee and staff would be pleased to take the suggestion to help people outside the hospital.

THE HOSPITAL October 15, 1910.

